# Sleep

**Published:** 1861-03

**Authors:** 


**SLEEP.** By W. W. Hall, M.D. New-York: Published by the Author.

The publications of Dr. Hall are known throughout the country, no less than in the Canadas and England. He is editor of Hall's Journal of Health, a monthly that is extensively transferred into the columns of newspapers and periodicals of the day. He is a physician in large practice; is a man of wide observation and experience; is intelligent and conscientious, and is exerting a great influence on the public mind in this country, in relation to the improvement of the public health. His teachings are practical, and are conveyed in a language that can be understood by all. It is our deliberate conviction that he is doing more work in reforming the unhealthful habits and practices of the people than any other man in the country.

His present work is a fit companion to those which have preceded it. It is chiefly devoted to the importance and necessity of sleeping in thoroughly pure air. In the language of the author, the "aim and end of the book is to show that as a means of high health, good blood, and a strong mind to old and young, sick or well, each one should have a single bed in a large, clean, light room, so as to pass all the hours of sleep in a pure fresh air, and that those who fail in this, will in the end fail in health and strength of limb and brain, and will die while yet their days are not all told." In pursuance of this position, Dr. Hall proceeds to enlarge upon the great subject of Sleep, and shows beyond refutation that our sleeping-rooms are extremely imperfect in respect to ventilation and other essentials as to size, etc. It is properly argued that the apartment in which we spend on an average one third of our existence, should be one in which pure air is freely admitted.

The adoption of Dr. Hall's teachings—and that they should be adopted we feel convinced—would bring about many changes in domestic life. The doctrine of single beds is philosophical, sanitary, common-senseful. We are glad that a man whose words are authority, has taken this subject in hand; and still more so that it has been treated in so thorough a manner. We cordially commend the work, which should go into every household in the land.—*Boston Atlas and Bee of Dec. 28th.*

"Allow me to express my gratification at having read it. You advocate very important reforms, and which the people will be compelled to consider sooner or later; the sooner the better. Of all the books you have written, none, as it seems to me, will do so much good in the end. I congratulate you sincerely on your success." P. C.

---

The above book, with Hall's Journal of Health, and also the Fireside Monthly for 1861, will be furnished for Three Dollars.

